# The hide and seek of *Plasmodium vivax* in West Africa: report from a large-scale study in Beninese asymptomatic subjects

**DOI:** 10.1186/s12936-016-1620-z

**Published:** 2016-11-25

**Authors:** Philippe Poirier, Cécile Doderer-Lang, Pascal S. Atchade, Jean-Philippe Lemoine, Marie-Louise Coquelin de l’Isle, Ahmed Abou-bacar, Alexander W. Pfaff, Julie Brunet, Lydia Arnoux, Elodie Haar, Denis Filisetti, Sylvie Perrotey, Nicodeme W. Chabi, Casimir D. Akpovi, Ludovic Anani, André Bigot, Ambaliou Sanni, Ermanno Candolfi

**Affiliations:** 1 Laboratoire de Parasitologie Mycologie, CHU Gabriel Montpied, Clermont-Ferrand, France; 2 Laboratoire Microorganismes: Génome et Environnement, UMR CNRS 6023, Aubière, France; 3 Institut de Parasitologie et de Pathologie Tropicale, EA 7292, Fédération de Médecine Translationnelle, Université de Strasbourg, Strasbourg, France; 4 Laboratoire de Biochimie et de Biologie Moléculaire, Université d’Abomey Calavi, Cotonou, Benin; 5 Laboratoire de Parasitologie et de Mycologie Médicale, Plateau Technique de Microbiologie, Hôpitaux Universitaires de Strasbourg, Strasbourg, France; 6 Agence Nationale pour la Transfusion Sanguine (Ministère de la Santé), Cotonou, Benin

**Keywords:** *Plasmodium vivax*, Malaria, ELISA, MSP1, CSP1, Western Africa, Benin, Blood donors

## Abstract

**Background:**

*Plasmodium vivax* is considered to be absent from western Africa, where the prevalence of Duffy-negative red blood cell phenotype proves to be high. Several studies have, however, detected *P. vivax* infection cases in this part of Africa, raising the question of what is the actual prevalence of *P. vivax* in local populations.

**Methods:**

The presence of *P. vivax* was investigated in a large population of healthy blood donors in Benin using microscopy, serology and molecular detection. The seroprevalence was measured with species-specific ELISA using two recombinant *P. vivax* proteins, namely r*Pv*MSP1 and r*Pv*CSP1. Specific molecular diagnosis of *P. vivax* infection was carried out using nested-PCR. The performances and cut-off values of both r*Pv*CSP1 and r*Pv*MSP1 ELISA were first assessed using sera from *P. vivax*-infected patients and from non-exposed subjects.

**Results:**

Among 1234 Beninese blood donors, no parasites were detected when using microscopy, whereas 28.7% (354/1234) of patients exhibited had antibodies against r*Pv*MSP1, 21.6% (266/1234) against r*Pv*CSP1, and 15.2% (187/1234) against both. Eighty-four samples were selected for nested-PCR analyses, of which 13 were positive for *P. vivax* nested-PCR and all Duffy negative.

**Conclusion:**

The results of the present study highlight an unexpectedly high exposure of Beninese subjects to *P. vivax*, resulting in sub-microscopic infections. This suggests a probably underestimated and insidious parasite presence in western Africa. While the vaccination campaigns and therapeutic efforts are all focused on *Plasmodium falciparum*, it is also essential to consider the epidemiological impact of *P. vivax*.

**Electronic supplementary material:**

The online version of this article (doi:10.1186/s12936-016-1620-z) contains supplementary material, which is available to authorized users.

## Background

About half of the world population is exposed to malaria parasites, and in 2015, the World Health Organization estimated that there were 214 millions of new cases of malaria worldwide, resulting in 438,000 deaths, essentially involving young African children [1]. While six parasitic species can cause human malaria, namely *Plasmodium falciparum*, *Plasmodium vivax*, *Plasmodium ovale curtisi*, *Plasmodium ovale wallikeri*, *Plasmodium malariae* and *Plasmodium knowlesi*, it must be stressed that *P. falciparum* and *P. vivax* are the most prevalent malaria parasite species. Whereas both species display a widespread distribution, *P. vivax* has a wider distribution than *P. falciparum*, having recently re-emerged in the south of Europe [2]. In a recent review, Moreira et al., have highlighted the substantial morbidity and mortality associated with *P. vivax* infection, along with its low parasitaemia in endemic countries, and in particular Southeast Asia, western Pacific, and South America [3]. While the overall *P. vivax* prevalence in Africa remains low, the parasite is considered to be present in the Horn of Africa, yet almost absent in West Africa. To date, this has been mainly accounted for by the absence of the red blood cell surface Duffy antigen among Africans living in this area [4]. Meanwhile, however, *P. vivax* infections were documented in Duffy-negative subjects in Brazil [5, 6], Ethiopia [7, 8], Madagascar [9], but also in West African countries, such as Mauritania [10], Cameroon [11, 12], Equatorial Guinea, and Angola [13]. According to these different studies, the prevalence of *P. vivax* in West-Africa is probably underestimated and large-scale epidemiological studies are thus required to investigate the burden of *P. vivax* infections [3].

Microscopy diagnostic methods have been shown to be time-consuming, requiring experienced personnel, while possibly leading to misidentification between *P. vivax* and *P. ovale* spp., which are highly prevalent in West Africa [14]. For epidemiological studies to assess *P. vivax* infections, molecular biology has been commonly used and shown to be more sensitive than microscopy [3, 15].

Molecular biology, however, remains expensive for many countries in endemic areas [16]. Serologic assays constitute a good alternative for epidemiological survey and blood donor screening, given that, antibodies reflect exposure to pathogens [17]. ELISA assays were recently developed for the detection of anti-*P. falciparum, P. ovale wallikeri/curtisi,* and *P. malariae* antibodies by using species-specific recombinant MSP1 proteins [18]. By this mean, authors of that previous study have been able to highlight an unexpected high seroprevalence of *P. ovale* spp. and *P. malariae* in Beninese blood donors.

For the present study, a specific *P. vivax* ELISA was developed using recombinant *P. vivax* merozoite surface protein 1 (r*Pv*MSP1) and circumsporozoite protein 1 (r*Pv*CSP1). Performance of this assay was first assessed in both French travellers positive for *P. vivax* and non-exposed-to-*Plasmodium* French blood donors. Then, the seroprevalence of *P. vivax* in an asymptomatic Beninese blood donor population was further explored for the first time.

## Methods

### Samples from *Plasmodium vivax* infected patients

Sera from 41 *P. vivax*-infected patients from French Guyana and Venezuela were used for the initial study. Diagnosis and species identification were carried out by means of the microscopical observation of blood smears and then confirmed by PCR, as previously described [19]. This population was employed in order to determine the sensitivity and positive predictive value of ELISA.

### Negative controls

Blood donor samples were collected at the Etablissement Français du Sang d’Alsace (EFS Alsace). Donors were classified as unexposed-to-malaria (280 samples) if their questionnaire responses indicated that they had never travelled to a malaria-endemic area. These samples were used to assess ELISA’s specificity and negative predictive value.

### Samples from Beninese blood donors

Plasma and total blood samples from blood donors without apparent malaria symptoms (n = 1234) were collected over 10 months (May 2009 to February 2010) in six Beninese departmental blood centres [20]. Each donor signed a consent form, and both the Direction of Benin National Blood Transfusion Agency and Research Ethics Committee of the Republic of Benin validated the protocol. This population had been previously studied in order to determine the specific seroprevalence of *P. falciparum*, *P. ovale* spp. and *P. malariae* in Benin using species-specific ELISA [18]. Quantification of circulating pLDH was also performed.

### Recombinant proteins

Nucleotide sequences encoding 203 AA (amino acids) of *P. vivax* merozoite surface protein 1 (r*Pv*MSP1; Accession number AAA63427.1) and 259 AA of *P.vivax* circumsporozoite protein 1 (r*Pv*CSP1; P08677.2) were cloned into expression vector pMAL-c2X (New England BioLabs, Ipswich, MA, USA). Both recombinant proteins were produced by *Escherichia coli* expression hosts and purified on amylose resin and DEAE Sepharose ^®^ (GE healthcare, Uppsala, Sweden).

### ELISA assays

Recombinant protein antibody screening was carried out using an in-house ELISA test derived from a commercial assay (DiaMed) [21]. r*Pv*MSP1 and r*Pv*CSP1 were immobilized overnight on 96-well plates at 4 °C in coating buffer and blocked for 1 h with phosphate buffered saline (PBST) with Tween 0.05% containing 1% bovine serum albumin (BSA) (Merck, Darmstadt, Germany). After washing with PBST, 200μL of diluent buffer (PBST with BSA 0.1%) were dispensed into each well and 10μL of serum incubated for 1 h at 37 °C. Using the same plate, 10 μL of positive and negative controls in triplicate were added. After three washes with PBST, 100 μL of horseradish peroxidase-labelled, monoclonal rabbit anti-human IgG (Sigma-Aldrich, St Quentin, France) were incubated for 30 min at 37 °C. After three washes with PBST, 100 μL of tetramethylbenzidine (TMB) plus substrate solution (Kem-en-tec, Denmark) were incubated for 15 min at 37 °C and the reaction was stopped with 50 μL of 0.5 M sulfuric acid. The absorbance was read within 30 min at 450 nm against 620 nm. Test validation required the positive OD to be >0.500 and the negative OD < 0.200. Average OD values for the 280 malaria-unexposed controls were 0.109 ± 0.095 and 0.112 ± 0.120 for r*Pv*MSP1 and r*Pv*CSP1, respectively. The cut-off value was set at three-fold the negative control wells’ average OD. The antibody (Ab) index of each sample was defined as ratio of its OD value and the cut-off value. The sample was considered positive if the Ab index was ≥1, and negative if the Ab index was <1. All assays were fully automated and performed on an EVOLIS Microplate System (Bio-Rad) at the Microbiology Department of the Hôpitaux Universitaires de Strasbourg.

### Blood DNA extraction

Total DNA from 1 mL of whole blood sample was extracted using the QIAmp Blood DNA mini kit^®^ (Qiagen, Hilden, Germany) according to the manufacturer’s recommendations and eluted in a final volume of 200 µL.

### Nested-PCR for the diagnosis and species identification of *Plasmodium* infection

Search for *Plasmodium* sp. using nested-PCR was conducted, as previously described [22]. Briefly, the reaction mix contained 1× GoTaq Green mix (Promega), 2 mM of MgCl_2_, 0.2 mM of dNTPs, 0.5 µM of primers, and 5 µL of DNA template (or PCR product) in a 50 µL final volume. First round of PCR was carried out using rPLU5 and rPLU6 and nested using rViv1 and rViv2 primers for detecting *P. vivax*, rFAL1 and rFAL2 for *P. falciparum*, rMAL1 and rMAL2 for *P. malariae*, rOVA1 and rOVA2 for *P. ovale curtisi* [22], and rOVA1v and rOVA2v for *P. ovale wallikeri* [23]. Amplification consisted in an initial denaturation step of 3 min at 95 °C, which was followed by 40 cycles of 30 s at 95 °C, and then 30 s at 58 °C, and thereafter of 1 min at 72 °C (30 s for the nested).

### Duffy genotype determination

Duffy genotype was assessed by sequencing the ~516 bp region of the gene encoding Duffy antigen as previously described [12, 24]. PCR products were sequenced by MWG Eurofins (Germany). Analysis of this sequence allows the T-33C mutation in the Duffy gene promoter to be detected. Single-nucleotide polymorphism (SNP) abolishes the expression of Duffy antigen in erythroid cells.

### Statistical analysis

Epidemiological data analyses were performed using the Chi squared test. Correlation coefficients were calculated using Pearson’s coefficients. Comparison between *r* ^*2*^ values was carried out using Williams’ *t* test for dependent data. A *p* value below 0.05 was considered statistically significant. Statistical analyses were conducted using GraphPad Prism^®^ software.

## Results

### Assessment of r*Pv*MSP1 and r*Pv*CSP1 ELISA

The assay sensitivity was first evaluated on 41 sera from *P. vivax*-positive febrile patients diagnosed using microscopy and then confirmed by species-specific PCR. Specificity was subsequently evaluated on 280 sera from non-exposed-to-malaria French blood donors. The r*Pv*MSP1 and r*Pv*CSP1 ELISA sensitivity values were 68.3 and 70.7%, respectively (Table 1). Specificity values were 99.3 and 96.8%, respectively, providing a better predictive positive value for r*Pv*MSP1 ELISA (93.3%) as compared to r*Pv*CSP1 (76.3%). Negative predictive values were similar between both assays, namely 95.5 and 95.8%, respectively. The test performance when combining the two ELISA (positive results for both r*Pv*MSP1 and r*Pv*CSP1, and negative if one of the two was negative) was also evaluated. When using this combination, sensitivity in *P. vivax* patients decreased to 53.7%, whereas both specificity and positive predictive values reached 100%, along with a 93.6% negative predictive value.

**Table 1:** Assessment of the antibody-ELISA using specific recombinant *Plasmodium vivax* proteins (r*Pv*MSP1 and r*Pv*CSP1)

*Plasmodium vivax*	r*Pv*MSP1 ELISA pos	r*Pv*CSP1 ELISA pos	r*Pv*MSP1 and r*Pv*CSP1 ELISA pos
Positive (n = 41)^a^	28/41	29/41	22/41
Negative (n = 280)^b^	2/280	9/280	0/280
Sensitivity	68.3%	70.7%	53.7%
Specificity	99.3%	96.8%	100.0%
Positive predictive value	93.3%	76.3%	100.0%
Negative predictive value	95.5%	95.8%	93.6%

^a^ *P. vivax*-infected travellers (confirmed by PCR)

^b^Blood donors without any travel history in malaria-endemic countries

### *Plasmodium vivax* seroprevalence study in Beninese blood donors

**Fig. 1:**
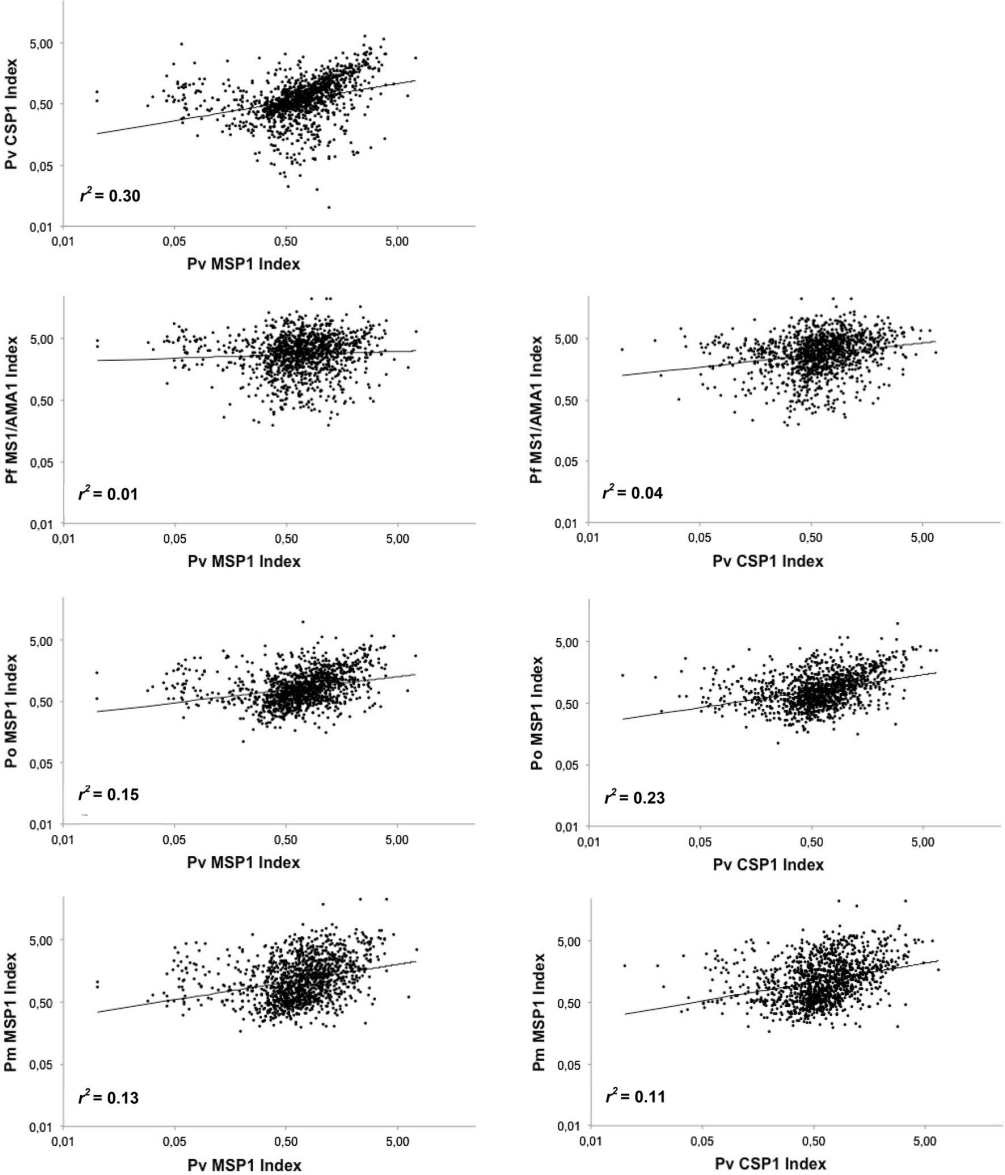
Linear regression analysis of the ELISA indexes from the different recombinant antigens within the 1234 Beninese blood donors. Correlation coefficient (*r* ^*2*^) was calculated using Pearson’s method

The recombinant antigens were used in order to evaluate *P. vivax* seroprevalence in a panel of 1234 Beninese blood donors. This cohort had previously been studied in order to assess the presence of antibodies directed against *P. malariae*, *P. ovale* spp. and *P. falciparum* species [18]. Anti-r*Pv*MSP1 antibodies were detected in 28.7% (354/1234) of the Beninese population, and anti-r*Pv*CSP1 in 21.6% (266/1234), with concomitant detection of both anti-r*Pv*MSP1 and anti-r*Pv*CSP1 in 15.2% of the cohort (187/1234). Data from a previous study were employed in order to determine the percentage of subjects also presenting with specific antibodies against other *Plasmodium* species [18, 21]. As shown in Table 2, many Beninese blood donors displayed antibodies against at least one other *Plasmodium* species. To evaluate possible cross-reactions between these malarial recombinant proteins, the correlation coefficient was calculated as previously described, and linear regressions were performed (Fig. 1) for the antigen pair indexes (r*Pv*MSP1/r*Pv*CSP1, r*Po*MSP1/r*Pv*MSP1, r*Po*MSP1/r*Pv*CSP1, r*Pm*MSP1/r*Pv*MSP1, r*Pm*MSP1/r*Pv*CSP1, r*Pf*MS1-r*Pf*AMA1/r*Pv*MSP1, r*Pf*MS1-r*Pf*AMA1/r*Pv*CSP1) [25]. There was a highly significant correlation between r*Pv*MSP1 and r*Pv*CSP1 indexes (*r* ^*2*^ = 0.3033; *p* < *0.05*; see Additional file 1: Table S1). While the correlation coefficients between other pairs were also significant, the r*Pv*MSP1/r*Pv*CSP1 *p* value proved to be the lowest (Additional file 1: Table S1). Thereafter, the *r* ^*2*^ value of the r*Pv*MSP1/r*Pv*CSP1 pair was compared to other pairs (Additional file 1: Table S1). Statistical analyses confirmed that r*Pv*MSP1/r*Pv*CSP1 *r* ^*2*^ value was significantly higher than that of all other pairs. Analyses of epidemiological data did not reveal any correlation between *P. vivax* infection and age. However, males were significantly more frequently seropositive for r*Pv*MSP1 than for r*Pv*CSP1 (Table 3), this difference between males versus females remaining significant even after combining both antigens.

**Table 2:** Seroprevalence study in Beninese blood donors using *Plasmodium* species-specific recombinant proteins

	Circulating pLDH	r*Pf*MSP1 + r*Pf*AMA1 pos^a^	r*Po*MSP1 pos^b^	r*Pm*MSP1 pos^c^	No other anti-*Plasmodium* antibodies^d^
r*Pv*MSP1 pos (n = 354)	17.8% (63/354)	75.4% (267/354)	61.0% (216/354)	75.4% (267/354)	2.3% (8/354)
r*Pv*CSP1 pos (n = 266)	25.6% (68/266)	97.4% (259/266)	67.3% (179/266)	82.0% (218/266)	0.4% (1/266)
r*Pv*MSP1 and r*Pv*CSP1 pos (n = 187)	19.6% (36/187)	77.0% (144/187)	71.7% (134/187)	84.5% (158/187)	0.5% (1/187)

^a^MSP1 and AMA1 *P. falciparum* recombinant proteins [18]

^b^ MSP1 *P. ovale* spp. recombinant protein [18]

^c^MSP1 *P. malariae* recombinant protein [18]

^d^Beninese blood donors without anti-r*Pf*MSP1, anti-r*Pf*AMA1, anti-r*Po*MSP1, anti-r*Pm*MSP1 antibodies

**Table 3:** Seroprevalence of *Plasmodium vivax* in males and females using ELISA detection of anti-r*Pv*MSP1 and/or anti-r*Pv*CSP1 antibodies

	Males (n = 988)	Females (n = 246)	*p* value
r*Pv*MSP1 pos (n = 354)	30.9% (305/988)	19.9% (46/246)	*0.0007*
r*Pv*CSP1 pos (n = 266)	22.4% (221/266)	18.3% (45/266)	0.1642
r*Pv*MSP1 and r*Pv*CSP1 pos (n = 187)	16.0% (158/988)	11.8% (29/266)	*0.0329*

Significant *p* values are in italics (*p* < 0.05)

### *Plasmodium vivax* detection by nested-PCR in Beninese blood donors

**Fig. 2:**
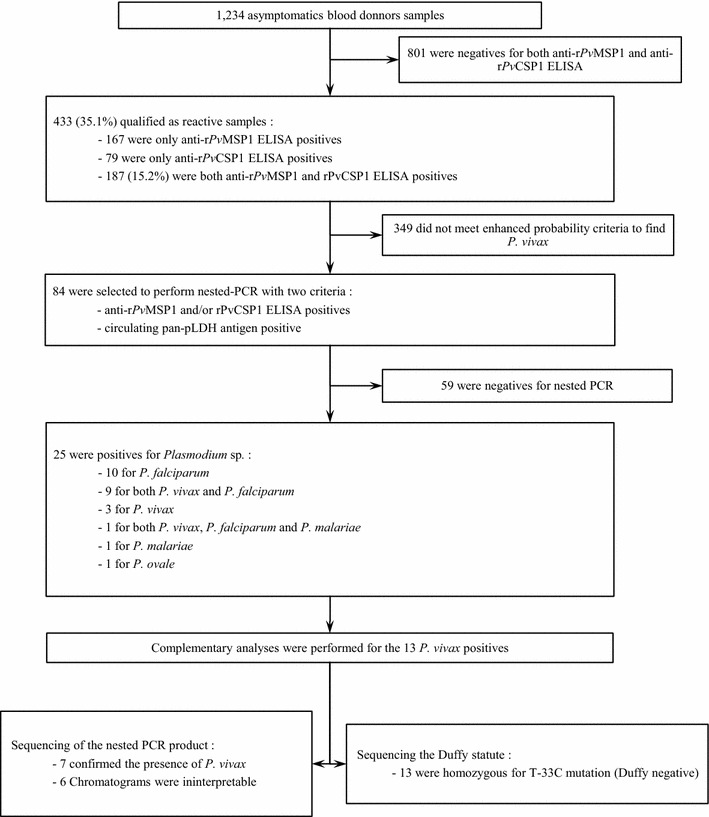
Sample flow diagram demonstrating progress through the study

Direct research of *P. vivax* was performed using nested-PCR, targeting a small region (~120 bp) of the ssrRNA gene [22, 23]. To enhance the probability to found *P. vivax*-positive subjects, blood samples (see flow diagram, Fig. 2) that were positive for circulating pan-pLDH antigen, with positivity for either r*Pv*MSP1 or r*Pv*CSP1 ELISA or for both, were selected. A total of 84 samples fulfilled these criteria, of which 25 were positive for *Plasmodium* sp. using nested PCR with 20/25 *P. falciparum*, 1/25 *P. ovale* spp., 2/25 *P. malariae*, and 13/25 *P. vivax*. Nine subjects were simultaneously infected by both *P. vivax* and *P. falciparum*, and one by *P. falciparum*, *P. malariae*, and *P. vivax*. To confirm *P. vivax* detection, the 13 nested-PCR products were sequenced. Chromatograms were interpretable for seven of them, thereby confirming the presence of *P. vivax* (Additional file 2: Fig. S1. Accession numbers KY014285-KY014291).

### Duffy determination

Duffy statute of the 13 subjects with a positive *P. vivax*-nested-PCR was established by sequencing. Homozygous T-33C mutation was detected in all 13 subjects, thereby indicating that these subjects were Duffy-negative.

## Discussion

Over the last decade, a new vision of the transmission of *P. vivax* in Africa has emerged, with evidence of its presence in West Africa [26]. Likewise, the dogma of Duffy-negativity protecting effect against *P. vivax* infection has being challenged [26]. New *P. vivax* mapping has been recently proposed, highlighting numerous grey areas in the knowledge [26]. As a result, the real burden of *P. vivax* in Africa is still unknown. Different hypotheses may account for the partial view of *P. vivax* epidemiology in Africa, including the dogma of Duffy’s protectivity, low parasitaemia of *P. vivax* compared to *P. falciparum* and inappropriate diagnostic tools for accurate large-scale screenings [27]. Difficulties with microscopy diagnosis were encountered in the first studies using PCR, revealing the presence of *P. vivax* in western Africa, where it was supposed to be absent [26]. Moreover, microscopy was shown to require experienced operators in order to avoid misidentification between *P. ovale* spp. and *P. vivax* [14].

Regarding the epidemiology of *P. vivax* in Africa, symptomatic subjects probably represent only the tip of the iceberg. Given this background, studies including large cohorts of asymptomatic subjects, such as in the present work, will be likely prove in this research field. It should be acknowledged, however, that parasitaemia in asymptomatic carriers was shown to be very low, making the use of direct diagnosis tools for the detection of these cryptic infections very challenging [8]. Furthermore, both PCR and microscopy are not able to exclude previously-cured *P. vivax* infections. For these reasons, a species-specific ELISA assay was developed in the present study in order to determine the *P. vivax* seroprevalence in large-scale cohorts of Beninese healthy people.

Benin is located in the intertropical region of West Africa, its climate considered warm with four seasons, two dry and two rainy [20]. Given that major *Anopheles* species vectors of *P. vivax* are present, Benin meets all the required conditions for a potential constant parasite transmission [28, 29]. However, the prevalence of *P. vivax* in Benin has never been studied before. Recombinant proteins were produced using regions of *Pv*MSP1 and *Pv*CSP1. Both proteins have been previously studied for their immunogenicity properties, while representing potential vaccine targets [30].

Given that the present study sought to explore *P. vivax* presence in Beninese blood donors, ELISA’s cut-off values were adjusted to favour the test’s specificity and positive predictive values. Sensitivities of this assays were approximately 70%, which may be accounted for by using the sera from travellers suffering from acute infection while having not yet produced antibodies against *P. vivax*, as previously reported for *P. falciparum* [21]. *Plasmodium vivax* seroprevalence in Beninese blood donors ranged from 28.7 to 15.2% when considering only either r*Pv*MSP1 results or the combination with r*Pv*CSP1. The present study was the first to investigate *P. vivax* presence in Benin, and only two prior studies investigated *P. vivax* seroprevalence in West Africa. Based on these study results, 13 and 5.2% of subjects from the Republic of the Congo and the Cameroon, respectively, exhibited antibodies specifically directed against specific *P. vivax* antigens [11, 25]. Results from the present study confirmed that the *P. vivax* seroprevalence in peoples living in West Africa amounts to at least 10%, if not more. In the present work, seroprevalence in males was significantly higher than in females, with the opposite being previously reported from the Republic of Congo [25], thereby pointing towards differences between the different areas as to the epidemiology and likely risk factors.

In a previous study, the same Beninese blood donor population was employed in order to assess species-specific ELISA for the diagnosis of *P. falciparum*, *P. ovale wallikeri*/*curtisi* and *P. malariae* [18]. While integrating these previous results into the present study, it was observed that most of the subjects with *P. vivax* antibodies were also reactive to one or more other *Plasmodium* species. Whereas significant correlation factors were observed between r*Pv*CSP1 or r*Pv*MSP1 indexes and other species-specific antigens, it was not consider this to correspond to cross-reactivity between epitopes. As a matter of fact, all Beninese blood donors were shown to live in co-endemic areas with active transmission of *P. falciparum*, *P. ovale* spp. and *P. malariae*. The same observation was made by Culleton et al., who concluded that this correlation was predictive of that exposure to infection by one malaria species would also be predictive of the risk as to exposure to another malaria species [25]. Moreover, statistical analyses confirmed that the correlation coefficient factor value of r*Pv*CSP1/r*Pv*MSP1 index was significantly higher than that of others. In order to definitively assess results from the present study, molecular diagnosis using nested-PCR was performed. As discussed above, antibody detection does not mean the parasite to be present in Beninese blood donors at the time of sampling. Subjects with a higher risk of having circulating *P. vivax* in their blood where therefore selected. Selection criteria were based on both r*Pv*CSP1 and r*Pv*MSP1 positive results, in association with the detection of pLDH circulating antigen. Among the 1234 Beninese blood donors, 84 fulfilled these criteria, of which 13 were positive for the *P. vivax*-nested-PCR. *Plasmodium falciparum* DNA was also detected in nine of them, with one subject being positive for *P. vivax*, *P. falciparum* and *P. malariae*. These results thus reinforce the hypothesis of multiple anti-*Plasmodium* sp. antibody detection among Beninese blood donors as DNAs from three different *Plasmodium* species may be present in the same individual. It has to be assumed that multiple plasmodial infections may be detected in the same subject/patient via serology screening and, therefore, that microscopy remains the gold standard diagnostic tool, which should be followed by species-specific PCR, and this especially for submicroscopic cases. However, present results also explain the difficulties associated with microscopy diagnosis of *P. vivax* in regions of high multiple endemic *Plasmodium* species. *Plasmodium vivax*-nested-PCR products sequencing confirmed the presence of *P. vivax*. While it has to be acknowledged that these short sequences did not enable further strain comparison, it must be stressed that it was impossible to amplify longer sequences, thereby reflecting the very-low parasitaemia in these samples.

Based on both serology and PCR results, the present study highlights an unexpected high prevalence of *P. vivax* in the Beninese population, whereas the Duffy-negative phenotype is expected to reach 98–100% [31]. Duffy phenotype was assessed in the 13 subjects with positive *P. vivax*-nested-PCR. All were Duffy-negative, confirming that the Duffy-negative status does not constitute a barrier for *P. vivax* infection, as previously reported [9]. This finding strongly suggests that *P. vivax* may be able to use alternative pathways so as to invade red blood cells [32].

Results from the present study, combined to previous ones, suggest that serological assays are probably the most powerful tools aimed to investigate the burden of *P. vivax* in western Africa. After the first studies providing irrevocable proof of the presence of *P. vivax* presence while using molecular tools on small targeted populations, large-scale investigations using serology are now required. Moreover, the present study demonstrates that molecular approaches do only detect a small number of carriers while widely underestimating the prevalence of *P. vivax*, especially in low endemic settings.

## Conclusion

*Plasmodium vivax* is now well-recognized to be involved in severe attacks and is able to develop treatment-resistance. While prophylactic and therapeutic efforts are rightly focused on *P. falciparum*, the epidemiological impact of *P. vivax* must thus also be taken into account. For *P. vivax*, given that transmission is generally lower, measuring transmission while using routine diagnosis tools may prove difficult. Molecular and serological methods are thus necessary in order to assess the real disease burden. The present results demonstrate that seroprevalence can be used as an additional tool to identify new areas of vivax malaria transmission. This information as well as the prevalence of all malaria species will likely prove essential for the successful implementation of malaria control and elimination programmes [27, 33].

## Additional files

**Additional file 1: Table S1.** Pairwise comparison of recombinant protein indexes using correlation coefficients (*r* ^*2*^).

**Additional file 2: Fig. S1.** Sequence alignment of the seven *Plasmodium vivax* sequence from nested-PCR. Sequence variations are highlighted by *black boxes.*
