# Supplementary material for: The hide and seek of Plasmodium vivax in West Africa: report from a large-scale study in Beninese asymptomatic subjects
Source: Malar J. 2016 Nov 25;15:570. doi: 10.1186/s12936-016-1620-z (PMC5123334; doi:10.1186/s12936-016-1620-z)
Supplement: Supplementary file 1 — Additional file 1: Table S1. Pairwise comparison of recombinant protein indexes using correlation coefficients (r 2). [file 12936_2016_1620_MOESM1_ESM.docx]

**Table S1 Pairwise comparison of recombinant protein indexes using correlation coefficients (*r^2^*).**

| Pairs of recombinant antigens | Correlation coefficient^*^ | | *Comparison of r^2^values*  *to* r*Pv*CSP1/r*Pv*MSP1 *r*^2^ value^†^ |
| --- | --- | --- | --- |
|  | *r^2^* | *p-value^‡^* | *p-value^‡^* |
| r*Pv*CSP1/r*Pv*MSP1 | 0.3033 | *<0.0001* | / |
| r*Pv*MSP1/r*Pf*MS1+r*Pf*AMA1 | 0.0144 | *<0.0001* | *<0.0001* |
| r*Pv*MSP1/r*Po*MSP1 | 0.1548 | *<0.0001* | *<0.0001* |
| r*Pv*MSP1/r*Pm*MSP1 | 0.1297 | *<0.0001* | *<0.0001* |
| r*Pv*CSP1/r*Pf*MS1-r*Pf*AMA1 | 0.0442 | *<0.0001* | *<0.0001* |
| r*Pv*CSP1/r*Po*MSP1 | 0.2331 | *<0.0001* | *0.0073* |
| r*Pv*CSP1/r*Pm*MSP1 | 0.1138 | *<0.0001* | *<0.0001* |
| ^*^Correlation coefficient was calculated using Pearson coefficient (r).  ^†^Correlation coefficients of each pair of recombinant antigens were compared to the correlation coefficient of r*Pv*CSP1/r*Pv*MSP1 pair.  ^‡^*p-values <0.05* were considered as significant | | | |
