# Supplementary material for: The hide and seek of Plasmodium vivax in West Africa: report from a large-scale study in Beninese asymptomatic subjects
Source: Malar J. 2016 Nov 25;15:570. doi: 10.1186/s12936-016-1620-z (PMC5123334; doi:10.1186/s12936-016-1620-z)
Supplement: Supplementary file 2 — Additional file 2: Fig. S1. Sequence alignment of the seven Plasmodium vivax sequence from nested-PCR. Sequence variations are highlighted by black boxes. [file 12936_2016_1620_MOESM2_ESM.docx]

BBD_2159_(KY014291) ----------------------------------------------------TTTTGCTA

BBD_757_(KY014285) TCGCTTCTAGCTTAATCCACATAACTGATACTTCGTATCGACTTTGTGCGCATTTTGCTA

BBD_1002_(KY014286) -----------------------------------------CTTTGTGCGCATTTTGCTA

BBD_1198_(KY014289) ----------------------------------------ACTTTGTGCGCATTTTGCTA

BBD_1161_(KY014288) ----------------------------------------ACTTTGTGCGCATTTTGCTA

BBD_1048_(KY014287) ----------------------------------------ACTTTGTGCGCATTTTGCTA

BBD_1235_(KY014290) ----------------------------------------ACTTTGTGCG-ATTTTGCTA

********

BBD_2159_(KY014285) TTATGTGTGTCTTTTAATTAAAATGATTCTTGTGACGGGCTTTCCTTGCCCCGGCTTGGA

BBD_757_(KY014285) TTATGTGT-TCTTTTAATTAAAATGATTCTTTTTAAGGACTTTCTTTGCTTCGGCTTGGA

BBD_1002_(KY014286) TTATGTGT-TCTTTTAATTAAAATGATTCTTTTTAAGGACTTTCTTTGCTTCGGCTTGGA

BBD_1198_(KY014289) TTATGTGT-TCTTTTAATTAAAATGATTCTTTTTAAGGACTTTCTTTGCTTCGGCTTGGA

BBD_1161_(KY014288) TTATGTGT-TCTTTTAATTAAAATGATTCTTTTTAAGGACTTTCTTTGCTTCGGCTTGGA

BBD_1048_(KY014287) TTATGTGT-TCTTTTAATTAAAATGATTCTTTTTAAGGACTTTCTTTGCTTCGGCTTGGA

BBD_1235_(KY014290) TTATGTGT-TCTTTTAATTAAAATGATTCTTTTTAAGGACTTTCTTTGCTTCGGCTTGGA

******** ********************** * * ** ***** **** *********

BBD_2159_(KY014291) AGTAAGGACTTTCTTTGCTTCGGCTTGGAAGT

BBD_757_(KY014285) AGTA----------------------------

BBD_1002_(KY014286) AGATA---------------------------

BBD_1198_(KY014289) AGT-----------------------------

BBD_1161_(KY014288) AGT-----------------------------

BBD_1048_(KY014287) AGTA----------------------------

BBD_1235_(KY014290) AGT-----------------------------
